# Carbon dioxide-boosted growth of high-density and vertically aligned carbon nanotube arrays on a stainless steel mesh†

**DOI:** 10.1039/d2ra04822a

**Published:** 2022-12-05

**Authors:** Jun Jie Cao, Yu Jiang, Hang Zhan, Yu Zhang, Jian Nong Wang

**Affiliations:** School of Mechanical and Power Engineering, East China University of Science and Technology 130 Meilong Road Shanghai 200237 China jnwang@ecust.edu.cn +86-21-64252360

## Abstract

Vertically aligned carbon nanotubes (VACNTs), a unique group of highly aligned CNTs normal to a substrate, have been extensively studied during the past decades. However, it is a long-standing challenge to improve the height of VACNTs due to the incidental deactivation of catalysts during growth. Herein, we demonstrate a facile strategy toward synthesizing high-density and well-aligned CNT arrays from *in situ* formed Fe-based catalysts on a stainless steel (SS) mesh. These catalysts were generated by direct oxidation–reduction treatment to the SS, which had excellent adhesion on the mesh substrate, and thus suppressed catalyst aggregation and promoted CNT growth under the flow of C_2_H_2_. In particular, by feeding additional CO_2_ at an optimal rate, the height of CNT arrays could be boosted from *ca.* 15 μm to *ca.* 80.0 μm, one of the highest heights observed for VACNTs on SS-based substrates so far. This is attributed to the prolonged activity of the catalysts by CO_2_ induced removal of extra carbon. Our study might provide an insight into the development of efficient strategies for VACNT growth on conductive substrates.

## Abstract 2

Vertically aligned carbon nanotubes (VACNTs), a unique group of highly aligned CNTs normal to a substrate, have been extensively studied during the past decades.

## Graphical abstract

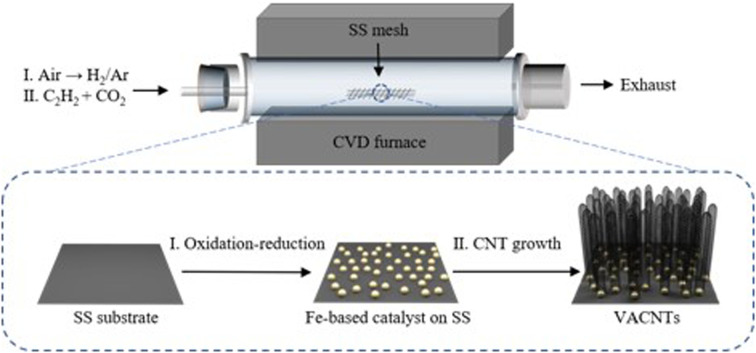

## 1 Introduction

Carbon nanotubes (CNTs) as a representative one-dimensional (1D) carbon material^1^ have been extensively studied due to their unique mechanical,^2^ electrical,^3^ thermal,^4^ and optical^5^ properties. For practical applications, it is necessary to assemble individual CNTs into macrostructures including CNT fibers^6^ and film.^7^ However, these macroscopic assemblies generally suffer from poor alignment of CNTs and low purity with residual catalysts, which seriously decays the mechanical or physicochemical properties of CNTs. In comparison, CNT arrays,^8^ a special class of highly aligned CNTs, which are bonded to each other *via* van der Waals forces, possess higher purity, significantly improved alignment and increased specific surface area. In particular, the vertically aligned CNT arrays (VACNTs)^9^ have achieved yields of several orders of magnitude higher (*e.g.*, ∼10^9^–10^13^ cm^−2^, quantified by the number density of CNTs on a substrate) than that of horizontally aligned CNT arrays (HACNTs, ∼10^4^ cm^−2^), thus showing great potential in a wide range of applications.^10–15^

Catalytic chemical vapor deposition (CCVD) has been one of the most commonly utilized approaches for preparing CNT arrays because of its simple operation, low cost, and relatively high growth efficiency.^16^ Up to date, VACNTs with millimeter-scale heights has been typically achieved by depositing catalysts on SiO_2_/Si substrates with an Al_2_O_3_ buffer layer.^17^ However, it is a tedious process to prepare the catalysts and then spray or sputter them on substrates. In addition, such VACNTs are subjected to limited applications due to the insulating or rigid properties of the substrates involved.

In recent years, using stainless steel (SS) as a conductive and flexible substrate has attracted some research.^18–21^ It has advantages of easy availability at low cost and high melting point (∼1300 °C), which enables the synthesis of CNTs at a wide temperature range. Furthermore, SS contains catalytic elements (*e.g.*, Fe), making it possible to achieve direct CNT growth without additional buffer layers or catalysts.^22^ For example, Camilli *et al.* demonstrated the SS surface with a nanoscale roughness can act as the catalyst/template for the CNT growth *via* the chemical vapor deposition (CVD) method.^23^ Thapa *et al.* reported the direct synthesis of VACNTs (10.7 μm in height) on a SS substrate by using plasma enhanced CVD without external catalyst layers.^24^ Although such direct VACNT growth on SS has been reported, issues remain regarding the deactivation of the catalysts, too early termination of CNT growth, and thus the short heights of the CNT arrays. Previous studies also reported that the addition of CO_2_ or H_2_O worked as a weak oxidant to prolong the catalyst lifetime. For example, Yang *et al.* found that the CO_2_-assisted floating-ferrocene CVD method is favorable for the well-controlled growth of aligned CNT arrays. CNTs grown with CO_2_ addition were also higher than those grown in the absence of CO_2_, with the optimum CO_2_ concentration of 760 ppm producing a 50% enhancement in CNT height.^25^ Sato *et al.* compared the CO_2_-assisted CVD with the H_2_O-assisted one for VACNT growth, and found that equivalent structures and yields were obtained in these two cases.^26^ However, the underlying mechanism has not been investigated. In fact, it is highly desired to induce persistent growth of VACNTs on SS substrates with improved heights for practical applications.

In this study, we report an effective method for facile growth of high-density and well-aligned CNT arrays on a SS mesh. Efficient catalyst particles are *in situ* constructed after the direct oxidation and subsequent reduction treatment to the SS mesh. This is beneficial for preventing the aggregation of the catalyst particles due to their strong interaction with the substrate of high-melting point. When acetylene (C_2_H_2_) is used as a reactive carbon feedstock, highly-aligned CNT arrays are grown with high density on the pre-treated SS mesh at the temperature of 700 °C. In addition, CO_2_ is employed as an additive to boost the growth of VACNTs to high heights by etching the amorphous carbon on catalysts and thus prolonging their lifetime.

## 2 Experimental methods

### 2.1 Preparation of VACNTs

A commercial SS mesh (316 grade) with 40 openings per inch (40 mesh) and a wire diameter of 190 μm was used as the substrate for VACNT growth. Prior to the usage, the SS mesh was cut into square sheets (1 cm × 20 cm) and then cleaned ultrasonically in acetone for 15 min and dried in air. The pre-cleaned SS mesh was placed at the center of a horizontal furnace equipped with a quartz tubular reactor (60 mm in diameter). Subsequently, it was heated from room temperature (*ca.* 25 °C) to 800 °C at a heating rate of 10 °C min^−1^ and then held for 30 min in the presence of air for the oxidization of the SS mesh. After that, the furnace was cooled to 700 °C in 10 min and gas lines were set up with the quartz reactor. A flow without CO_2_ as an additive was supplied at a flow rate of 10 sccm for the growth of VACNTs. Finally, the furnace was cooled down to room temperature under Ar flow (300 sccm), and the resultant samples were taken out for examination.

### 2.2 Structure characterization

Scanning electron microscope (SEM, Hitachi S3400, accelerating voltage of 15 kV) and transmission electron microscope (TEM, JOEL-2010F, accelerating voltage of 200 kV) were used for characterizing the structures and morphologies of the SS mesh and CNT samples. For TEM analysis, the CNTs were obtained by sonicating the VACNTs/SS mesh in ethanol and dripping the suspension liquid onto a TEM grid. Raman spectroscopy (Senterra R200-L, excitation wavelength of 532 nm, spatial resolution ≤ 1 μm, spectral resolution ≤ 1 cm^−1^) was used to evaluate the graphitization degree of the as-obtained VACNTs. The X-ray diffraction patterns were tested for 2*θ* from 10° to 80° at a scanning rate of 5° min^−1^ by an X-ray diffractometer (max2550VB, fit out with Cu Kα radiation, *λ* = 0.15406 nm). Gas chromatography (GC2060) was used to detect the compositions and concentrations of the exhaust from the tubular reactor.

## 3 Results and discussion

**Fig. 1:**
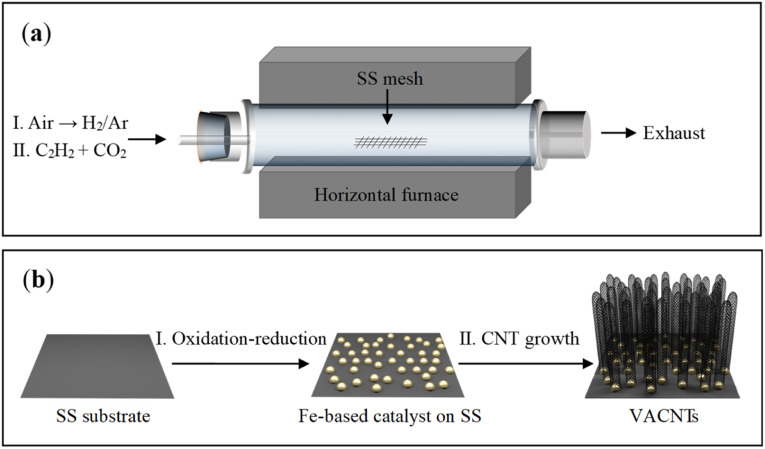
Schematic illustrations of the experimental setup (a) and the process for the growth of VACNTS on SS mesh (b).

The schematic diagram for growing VACNTs on the SS mesh is illustrated in Fig. 1a. Firstly, the SS mesh was annealed in air for initial oxidation. Compared with the relatively smooth surface of the pristine SS mesh (Fig. S1a and b†), nanoparticles with an average diameter of 300 nm were generated after the annealing treatment (Fig. S1c†). A flow of H_2_/Ar gas (10 vol% H_2_) was then fed into the quartz tube, giving rise to the formation of smaller nanoparticles as the reductive products (Fig. S1d†). Subsequently, C_2_H_2_ was fed as the highly reactive carbon precursor for VACNTs growth. As illustrated in Fig. 1b, the *in situ* formed nanoparticles on the SS mesh can work as catalytic sites for the nucleation of CNTs, which grew into VACNTs by squeezing each other along the direction perpendicular to the substrate under van der Waals force (Fig. 1b).

**Fig. 2:**
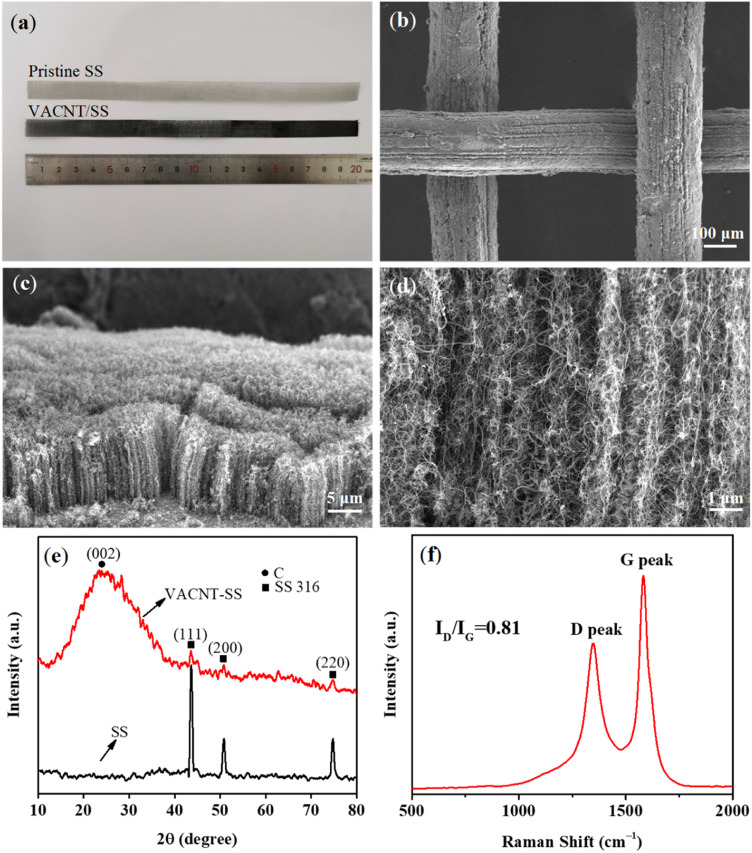
(a) Optical image of the pristine SS mesh and VACNTs/SS; (b–d) SEM images of VACNTs/SS at different magnifications; (e) XRD patterns for the pristine SS and VACNTs/SS; (f) Raman spectrum of the VACNTs/SS.

Fig. 2a presents the optical image of the pristine SS mesh and VACNTs grown on the pre-treated SS (VACNTs/SS). It is noticed that the as-obtained VACNTs/SS became visibly black, suggesting the successful growth of VACNTs on the SS mesh. The low-magnification SEM image (Fig. 2b) further shows the coverage of VACNTs on the SS mesh, demonstrating the uniform distribution of catalytic sites. Fig. S3† shows the elemental mapping of VACNTs/SS. As shown in Fig. 2c, the CNT arrays were dense and well-aligned and the average height could be determined to be *ca.* 15 μm. The high-magnification SEM image (Fig. 2d) shows that these CNTs were curly and combined with each other, but still along the direction perpendicular to the substrate. Fig. 2e shows the X-ray diffraction patterns of the SS mesh before and after the VACNTs growth. The peaks located at 44°, 51°, and 75° recorded from the pristine SS mesh can be assigned to the (111), (200), and (220) planes of Fe metal (PDF No. 65-9094), respectively. In comparison, the intensity of those peaks for VACNTs/SS was significantly decreased, along with the emergence of one distinct peak at 25° corresponding to the (002) plane of CNTs. These observations suggest that the SS mesh was well covered by dense and tall VACNTs, leading to the reduced detection depth of substrates and thus decreased peak intensity. In addition, the XRD results of the as-oxidized SS mesh and oxidation–reduction treated SS are illustrated in Fig. S2.† The main components of the as-oxidized SS mesh and oxidation–reduction SS mesh were Fe_2_O_3_ and Fe, respectively. Fig. 2f shows the Raman spectrum of VACNTs with a high ratio (0.81) of *I*_D_ (defect mediated D-band at ∼1350 cm^−1^) and *I*_G_ (graphitic nature G-band at ∼1590 cm^−1^), indicating good structural quality of the as-prepared VACNTs.

**Fig. 3:**
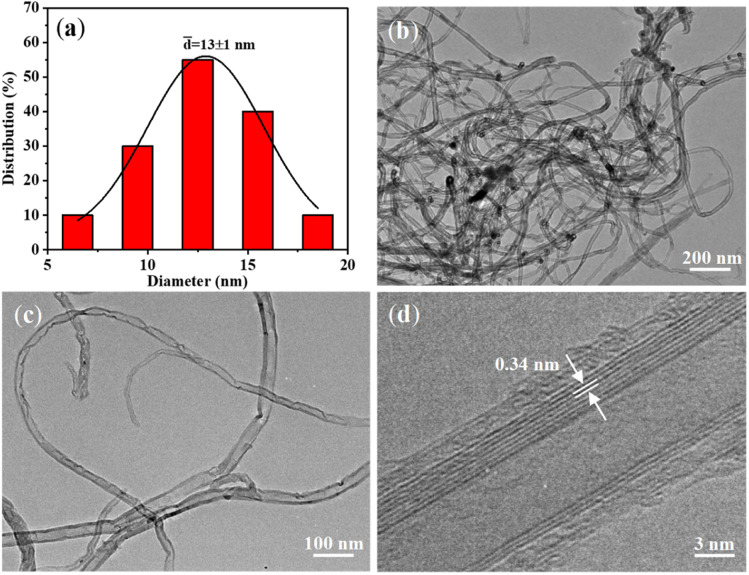
(a) Diameter distribution of the VACNTs; (b–d) TEM images of CNTs obtained by sonicating VACNTs/SS in ethanol.

The average diameter of CNTs was determined as *ca.* 13 nm (Fig. 3a) by statistical analysis from the low-magnification TEM image (Fig. 3b). The CNTs showed “bamboo-like” structures (Fig. 3c), which could be due to the dynamic changes of the catalyst particles in shape and size caused by the dissolution and precipitation of carbon atoms during the growth process. In addition, few catalyst particles were observed within or at the end cap of the CNTs, suggesting the base growth mode with catalysts anchored on the substrate during growth (Fig. S7†). This could be mainly due to the strong bonding of the *in situ* formed catalysts with the substrate by constructing chemical bonds during the oxidation–reduction process, which is favourable for preventing catalysts from migration and aggregation. In addition, the high-resolution TEM image demonstrates the multi-walled structure of the CNTs (Fig. 3d) with the clear *d*-spacing of 0.34 nm.

Previous studies have demonstrated that the growth time played a key role in improving the length of CNTs.^27–29^ The present growth duration was extended from 30 min to 2 h with the expectation of further improving the height of VACNTs. Although the flow rate of C_2_H_2_ as the carbon precursor was as low as 10 sccm, a large number of particles (Fig. S4a and b†) appeared on the top of VACNTs after 2 h of growth. This observation suggests that the catalysts had been gradually deactivated, leading to the covering of carbon atoms on VACNTs instead of continuous growth.

**Fig. 4:**
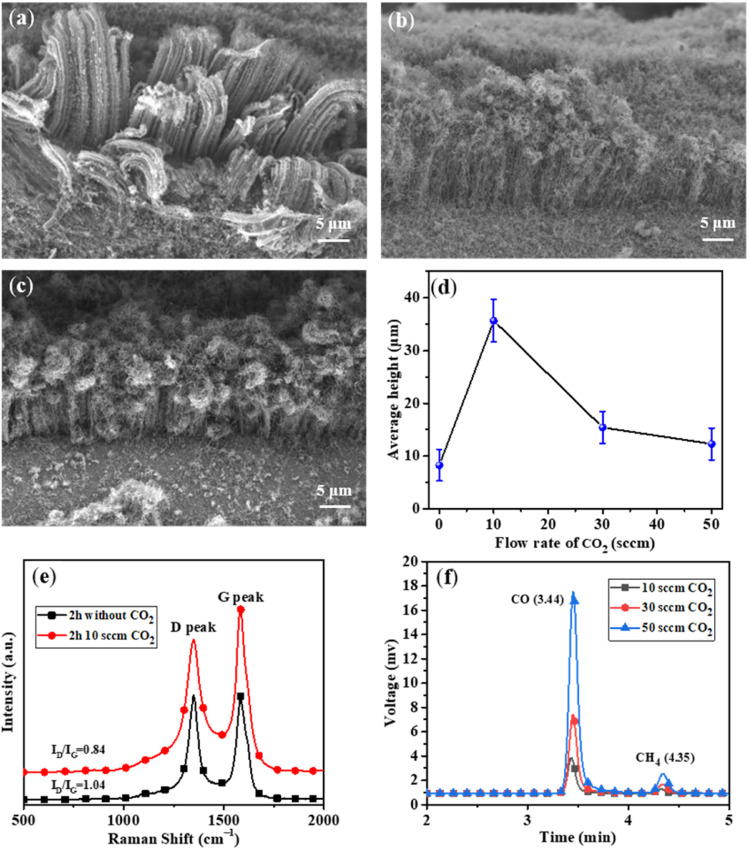
SEM images of VACNTs grown with the addition of CO_2_ at flow rates of 10 sccm (a), 30 sccm (b), and 50 sccm (c) in 2 h; (d) the average heights of VACNTs grown on SS mesh at different flow rates of CO_2_; (e) Raman spectra of VACNTs grown with CO_2_ (10 sccm) and without CO_2_ in 2 h; (f) gas chromatography of the exhaust at different flow rate of CO_2_.

**Fig. 5:**
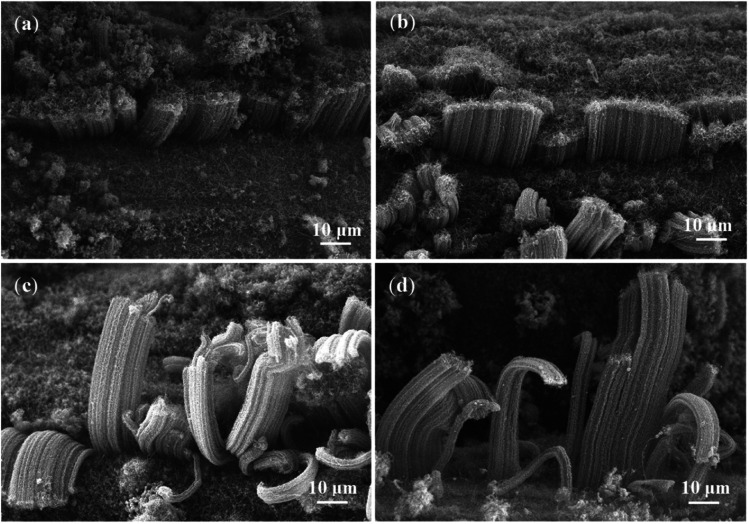
SEM images of VACNTs grown on SS with CO_2_ (10 sccm) in different growth durations: (a) 0.5 h; (b) 2 h; (c) 4 h; (d) 6 h.

In order to prolong the lifetime of the catalysts, CO_2_ was supplied as a weak oxidant by reacting with the carbon byproducts (*e.g.*, amorphous carbon) on the surfaces of the catalysts.^30–32^ The CNT growth was performed at different CO_2_ flow rates of 10, 30, and 50 sccm, while other experimental conditions were kept to be the same. As shown in Fig. 4a, the height of VACNTs was improved from ∼10 μm to ∼35 μm compared with that of the sample prepared without CO_2_, demonstrating the significant effect of CO_2_ on boosting the CNT growth. However, when the flow rate of CO_2_ was increased to 30 sccm (Fig. 4b) and 50 sccm (Fig. 4c), the top layer of VACNTs became intertwined and the length was gradually reduced (Fig. 4d). This could be attributed to the lowered activities of the catalyst nanoparticles in the presence of too much CO_2_. At the optimal flow rate of CO_2_ of 10 sccm, the growth time was increased 30 min to 6 h. As a result, the VACNTs with a maximum height of 80 μm was obtained (Fig. 5a–d). This height appears to be the highest among the VACNTs prepared with the use of SS as the substrate (Table S2†).

To explore the role of CO_2_ in CNT growth, the measurement of Raman spectroscopy was conducted for the samples obtained with or without CO_2_ feeding during the growth for 2 h. It was found that the *I*_D_/*I*_G_ value of VACNTs decreased from 1.04 to 0.84 with CO_2_ feeding (Fig. 4e), suggesting the effect of CO_2_ on improving the graphitization degree of VACNTs. This could be mainly ascribed to that CO_2_ at an optimal flow rate can properly etch the amorphous carbon on CNTs by following the reaction of CO_2_ + C → 2CO. The schematic illustrating the mechanism involved in the presence/absence of CO_2_ is shown in Fig. S5.† However, CO_2_ at higher concentrations may not only react with amorphous carbon but also oxidize the Fe-based catalysts, leading to their deactivation and thus inhibition of the decomposition of C_2_H_2_. The XRD measurement of VACNT/SS to characterize the components upon CO_2_ treatment is illustrated in Fig. S6.† It can be found that the three main peaks locating at 43.72°, 50.94° and 74.94° correspond to diffractions of (111), (200), and (220) planes of Fe, respectively, while the other minor peak at 35.48° can be assigned to (400) plane of Fe_3_O_4_. These results confirmed our suggestion that the catalysts for CNT growth were mainly Fe after the oxidation–reduction process, and excessive CO_2_ could also react with Fe and form Fe-oxides (Fe_3_O_4_).

To further explore the mechanism of CO_2_ in prolonging the life time of the catalysts, gas chromatography was used to detect the concentrations of CO in the exhaust when different flow rates of CO_2_ were used. As shown in Fig. 4f, the concentration of CO gradually increased with increasing flow rate of CO_2_ (Fig. 4f and Table S1†), verifying the etching effect of CO_2_ by reacting with amorphous carbon.

In addition, we further used a larger SS mesh (2 cm × 50 cm) as the substrate to grow VACNTs with the purpose of verifying the possibility of scaling up using the same procedure. As shown in Fig. S8a,† the entire substrate was observed to be covered uniformly with black materials. We also observed the growth of VACNTs at different positions of the substrate (Fig. S8b–d†), thus demonstrating that our method holds potential for large-scale production of VACNTs.

## 4 Conclusion

In summary, a facile strategy toward direct growth of high-density and well-aligned CNT arrays has been demonstrated by *in situ* constructing effective catalysts on a SS mesh. When the C_2_H_2_ gas at a flow rate of 10 sccm was used as the carbon source, VACNTs with an average height of 15 μm was obtained in 30 min, and this height didn't increase even when the growth time was extended. In contrast, with the inclusion of CO_2_ at a proper rate, VACNTs with a maximum height of 80 μm on the SS mesh were obtained with an extended growth duration. The effect of CO_2_ was clarified by both Raman spectroscopy and gas chromatography analysis, which may result from the prolongation of the activity of catalysts and thus the boosting of CNT growth. Considering the features of the present experiments, our work might provide an efficient and economic method for growing VACNTs on conductive substrates at large scale.

## Conflicts of interest

There are no conflicts to declare.

## Supplementary Material

RA-012-D2RA04822A-s001
