# Supplementary material for: Carbon dioxide-boosted growth of high-density and vertically aligned carbon nanotube arrays on a stainless steel mesh
Source: RSC Adv. 2022 Dec 5;12(53):34740–5. doi: 10.1039/d2ra04822a (PMC9720505; doi:10.1039/d2ra04822a)
Supplement: RA-012-D2RA04822A-s001 [file RA-012-D2RA04822A-s001.pdf]

Electronic Supplementary Material (ESI) for RSC Advances.
This journal is © The Royal Society of Chemistry 2022

Supporting information

Carbon dioxide-boosted growth of high-density and vertically aligned CNT arrays on a stainless steel mesh

Jun Jie Cao, Yu Jiang, Hang Zhan, Yu Zhang*, Jian Nong Wang*

School of Mechanical and Power Engineering East China University of Science and Technology, 130 Meilong Road, Shanghai 200237, China.

** Corresponding author. Tel: 86-21-64252360.*

E-mail: *jnwang@ecust.edu.cn (Jian Nong Wang)*

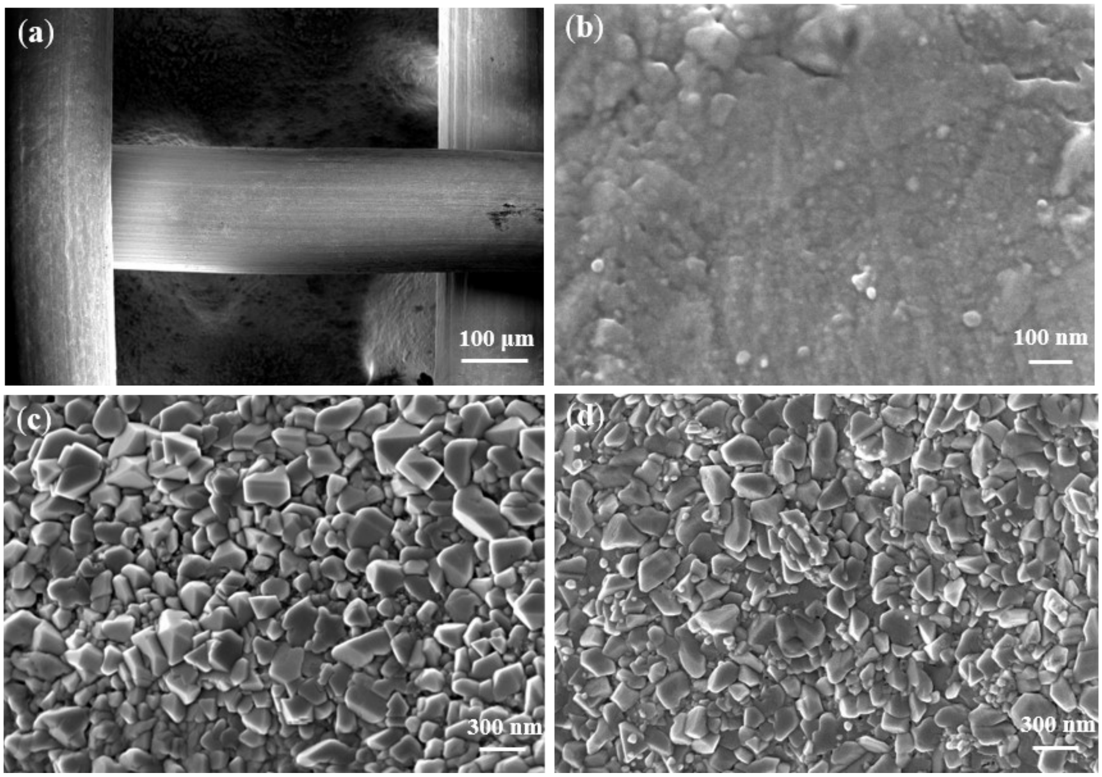

Fig. S1. Morphology evolution of the substrate surface. Pristine SS mesh at low (a) and high magnifications (b); (c) As-oxidized SS mesh; (d) Oxidation-reduction treated SS mesh

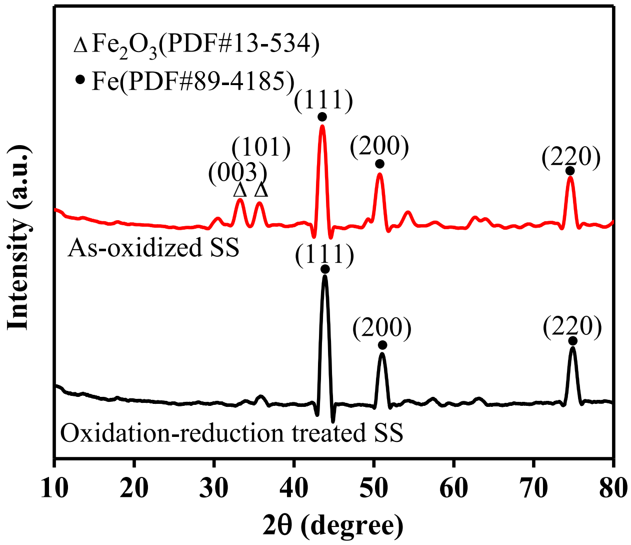

Fig. S2. XRD patterns for the as-oxidized SS mesh and oxidation-reduction treated SS

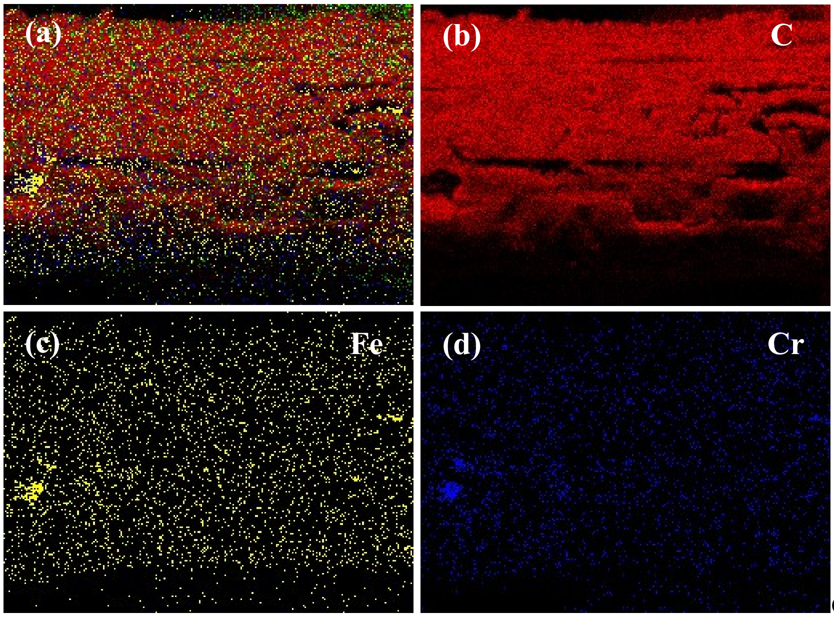

cc

Fig. S3. Elemental mapping of VACNTs/SS

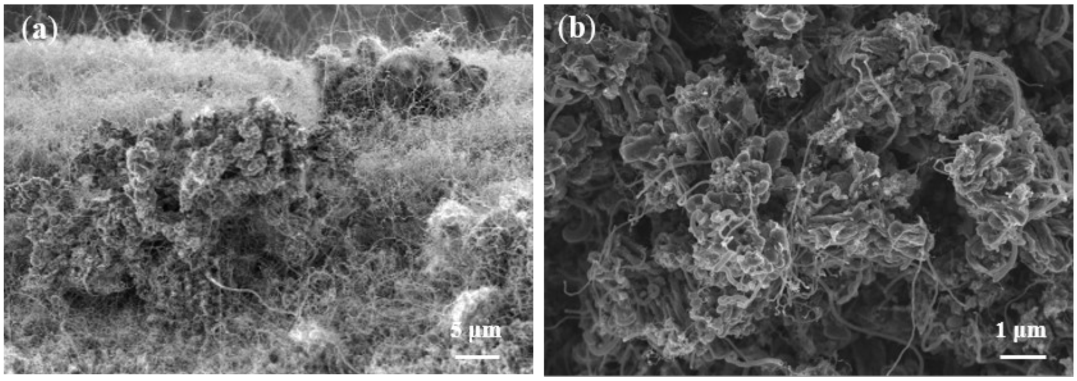

Fig. S4. SEM images at different magnifications recorded from the samples when the growth time was increased to 2 h, showing a large number of particles on the surface

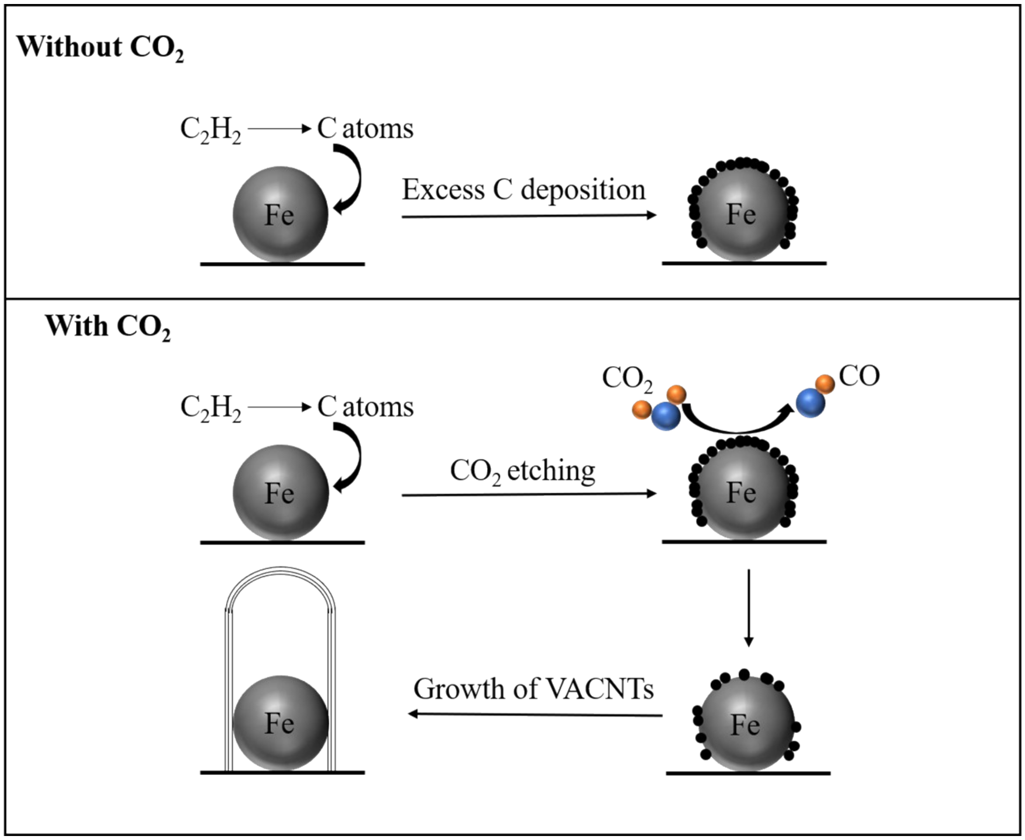

Fig. S5. A schematic illustraiting the mechanism involved in the presence/absence of CO_2

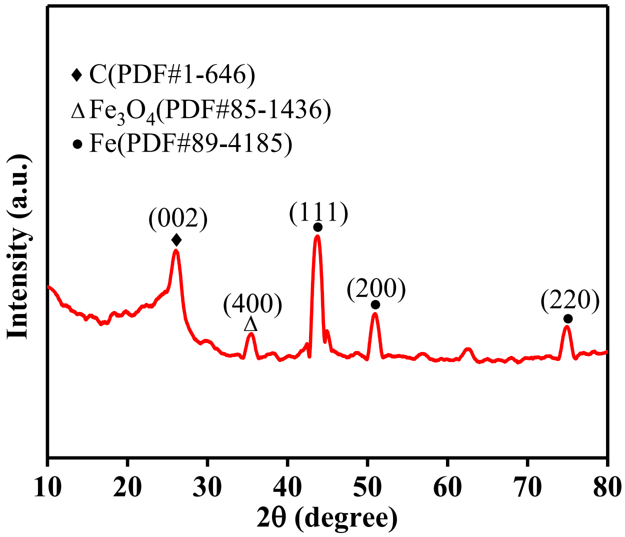

Fig. S6. The XRD measurement of VACNTs/SS to characterize the components upon

CO_2 treatment

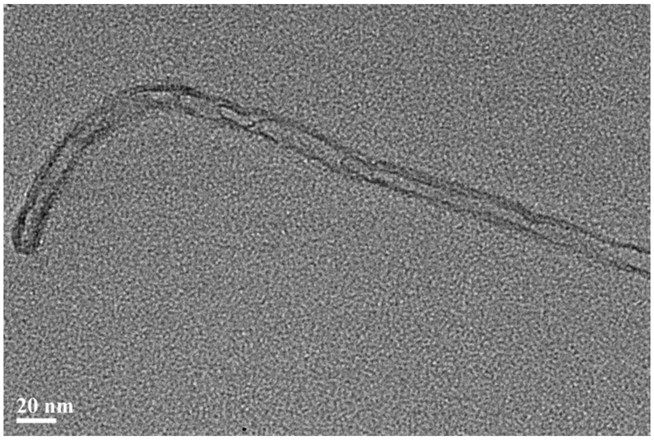

Fig. S7. The TEM image recorded from the tip of one typical CNT

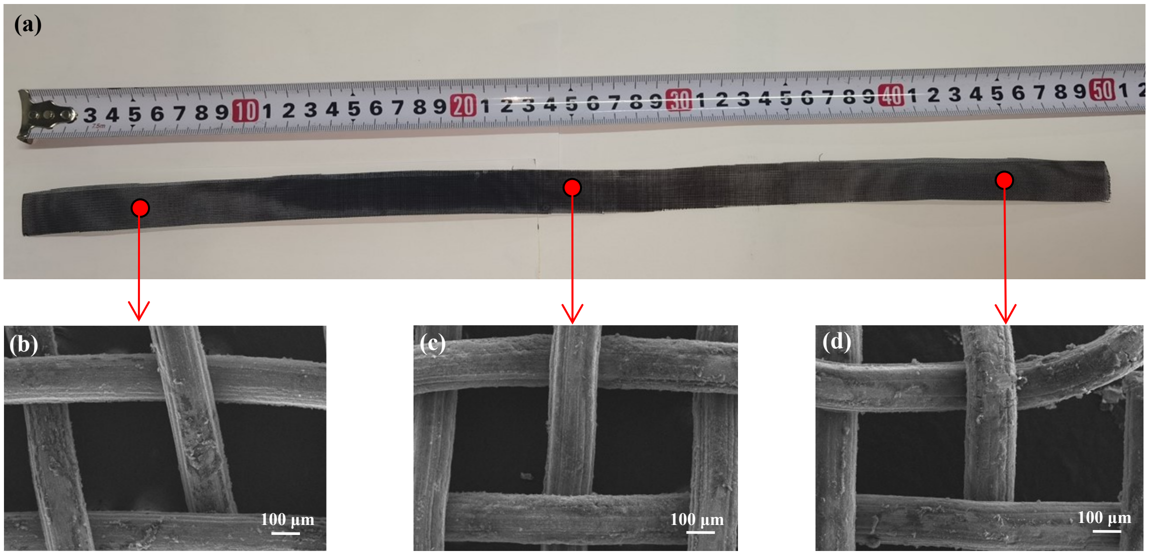

Fig. S8. A larger SS mesh (2 cm × 50 cm) as the substrate to grow VACNTs. (a) Optical image; (b-d) SEM images of VACNTs grown at different positions.

Table S1. GC results of the exhaust at different flow rates of CO_2

Flow rate of CO_2 (sccm)	Residue time (min)	Peak height (mv)	Peak area (mv·s)	Concentration of CO (ppmv)
10	3.44	2.929	18372.9	39.4027
30	3.45	6.448	39788	84.2722
50	3.45	16.534	109609	230.5631

Table S2. Comparison of VACNT heights on different conductive substrates

Substrate	Method	Carbon source	Temperature (°C)	Height (μm)	Ref.
SS 316	oxidation-reduction with CO_2 addition	C_2H_2	700	80	This study
SS 316	oxidation-reduction	C_2H_4	700	25	1
SS 304	oxidation-reduction	C_2H_2	700	15	2
SS 304	etching in HCl (35%)	C_2H_2	700	31	3
SS 304	etching in HCl (38%)	C_2H_2	700	40	4
SS 304	PECVD	C_2H_2	800	8	5
Al	ultrasonic spray	C_2H_2	620	5	6
Al	laser etching	C_2H_2	600	10	7
W	sputtering	C_2H_2	625	20	8
Ti	sputtering	C_2H_4	700	40	9

References

1 N. Sano, T. Kodama and H. Tamon, *Carbon*, 2013, **55**, 365.

2 H. Wang and C. Na, *ACS Appl. Mater. Interfaces*, 2014, **6**, 20309.

3 P. Avasthi, A. Kumar and V. Balakrishnan, *ACS Appl. Mater. Interfaces*, 2019, **2**, 1484.

4 C. E. Baddour, F. Fadlallah, D. Nasuhoglu, R. Mitra, L. Vandsburger and J.-L. Meunier, Meunier, *Carbon*, 2009, **47**, 313.

5 A. Thapa, S. Neupane, R. Guo, K. L. Jungjohann, D. Pete and W. Li, *Diam. Relat. Mater.*, 2018, **90**, 144.

6 I. B. Dogru, M. B. Durukan, O. Turel and H. E. Unalan, *Prog. Nat. Sci.*, 2016, **26**, 232.

7 S. Huang, X. Du, X. Li, M. Ma and L. Xiong, *Adv. Funct. Mater.*, 2021, **31**, 2104531.

8 P. M. Parthangal, R. E. Cavicchi and M. R. Zachariah, *Nanotechnology*, 2007, **18**, 185605.

9 A. Szabó, P. Andricević, Z. Pápa, T. Gyulavári, K. Németh, E. Horvath, L. Forró and K. Hernadi, *Front. Chem.*, 2018, **6**, 593.
